# Emergency inguinal hernia repair under local anesthesia: a 5-year experience in a teaching hospital

**DOI:** 10.1186/s12871-016-0185-2

**Published:** 2016-03-19

**Authors:** Tao Chen, Yunhe Zhang, Haolu Wang, Qihong Ni, Linhua Yang, Qiwei Li, Jian Wang

**Affiliations:** 1 Department of Biliary-pancreatic Surgery, Ren Ji Hospital, School of Medicine, Shanghai Jiao Tong University, 1630 S. Dongfang Road, Shanghai, 200127 China; 2 Therapeutics Research Centre, School of Medicine, The University of Queensland, Princess Alexandra Hospital, Woolloongabba, QLD 4102 Australia

**Keywords:** Incarcerated inguinal hernia, Local anesthesia, Safety, Effective

## Abstract

**Background:**

Local anesthesia (LA) has been reported to be the best choice for elective open inguinal hernia repair because it is cost efficient, with less post-operative pain and enables more rapid recovery. However, the role of LA in emergency inguinal hernia repair is still controversial. The aim of this study is to investigate the safety and effectiveness of LA in emergency inguinal hernia repair.

**Methods:**

All patients underwent emergency inguinal hernia repair in our hospital between January 2010 and April 2014 were analyzed retrospectively in this study. Patients were divided into LA and general anesthesia (GA) group according to the general conditions of the patients decided by anesthetists and surgeons. The outcome parameters measured included time to recovery, early and late postoperative complications, total expense and recurrence.

**Results:**

This study included a total of 90 patients from 2010 to 2015. 32 patients (35.6 %) were performed under LA, and 58 (64.4 %) were performed under GA. LA group has less cardiac complications (*P* = 0.044) and respiratory complications (*P* = 0.027), shorter ICU stay (*P* = 0.035) and hospital stay (*P* = 0.001), lower cost (*P* = 0.000) and faster recovery time (*P* = 0.000) than GA group.

**Conclusion:**

LA could provide effective anesthesia and patient safety in emergency inguinal hernia repair.

## Background

With progresses in surgical techniques and anesthetic methods, elective inguinal hernia repair surgery has become a safe outpatient procedure that carries favorable outcomes [1]. However, when it comes to emergency hernia repair surgery, things are different. Compared with elective surgery, postoperative mortality can increase 7-fold in emergency operations, and 20-fold if bowel resection was undertaken [2]. With fewer preoperative preparations and more difficult local anatomy, these patients are more likely at high risk of postoperative complication, or even death [3, 4].

Local anesthesia (LA) is one of the most commonly used anesthetic methods in inguinal hernia repair [5–7]. LA is recommended for inguinal hernia repair in elderly patients and patients with co-morbidities (grade C) by the Association of Surgeons of Great Britain and Ireland (ASGBI) [8]. Moreover, European Hernia Society Guideline (EHS) recommended that patients with ASA (American Surgical Association) preoperative evaluation of grade 3 or 4 can consider day surgery with LA. However, young anxious patients, with morbid obesity, incarcerated hernia should be excluded from operation under LA [9].

From 2008, our hospital started to perform inguinal hernia repair under LA. Compared with general anesthesia (GA), LA showed increased safety, better postoperative pain control, less postoperative complication, shorter recovery period, and reduced cost. Since 2010, our hospital has begun to perform emergency hernia surgery under LA. The aim of this study is to investigate the safety and effectiveness of LA in an emergency inguinal hernia surgery by evaluating related outcomes.

## Method

This is a retrospective study. We included all patients diagnosed as an incarcerated inguinal hernia and underwent an emergency inguinal hernia repair surgery in our hospital between January 2010 and April 2014. All the patients’ information required was collected from electronic medical records. The collection and analysis were approved by the Ethics Committee of Ren Ji hospital.

### Surgeons and surgical procedures

Emergency inguinal hernia repairs under LA were performed by two experienced attending surgeons who had performed at least 50 elective cases before. Operations under GA were performed by other three experienced attending surgeons in our department.

Data of patients were excluded if the contents of hernia sacs returned spontaneously after the anesthesia before operation started. There was no other exclusion criterion in this study. If no bowel resection was performed, tension-free mesh repair were employed. If strangulated hernia was clearly diagnosed, tissue of strangulation was needed to be removed and non-mesh repair was used to avoid the high risk of infection after bowel resection.

Cefotetan was used as prophylactic antibiotics routinely. Aztreonam was utilized if patients were allergic to cephalosporins.

### Anesthesia

The selection of anesthesia means (LA or GA) was decided by anesthetists and surgeons according to the general conditions of the patients.

In group GA, anesthesia was induced with propofol 2 mg/kg and fentanyl 0.1–0.2 mg intravenously. Inhalation anesthesia was given at the same time with a mixture of oxygen and isoflurane 1–2 % through an intubation.

Patients in LA group received the local infiltration technique. A mixture of 2 % lidocaine 20 ml and 0.9%NS 30 ml was used as the local anesthetic. Patients required extra analgesia during the surgery were given 20–40 mg parecoxib sodium intravenously. Conversion to GA was performed if LA was intolerant for patient, which was evaluated by both anesthetists and surgeons.

Patients in both groups were prescribed tramadol 100 mg per 24 h for the first 1–2 days postoperative.

### Postoperative recovery

In this study, “time to eat” is the post-operative time when patients were able to have semi-liquid without an intolerable nausea or vomiting. Patients were also asked to get up from the bed and walk a standard distance before they returned to their bed as soon as possible after the operation, which was recorded as “time to ambulation”.

Patients were required for a regular follow-up in our outpatient clinic after discharge (7, 30 and 90 days post-operatively), where all complications, including wound complications, scrotal edema, retention of urine and recurrence, were recorded.

### Economics

A data collection form recording all aspects of hospital resources was used in the study. It included the data on operation and staff cost, costs of hospital stay (including stay in ICU), and other healthcare costs, including cost of complications.

### Statistical analysis

Statistical analysis was performed using SPSS 19 software. Between-group differences were analyzed using two-sample t-test. When there was doubt about the validity of parametric assumptions, nonparametric tests were performed. A chi-square test or Fisher’s Exact Test, as appropriate, was used to analyze categorical data. A significance level of 0.05 was used in this study.

## Result

In this study, patients who underwent emergency hernia repair between January 2010 and April 2014 were included. 92 patients were enrolled initially. Two were excluded because contents of hernia sacs returned spontaneously after the anesthesia before the operation started (one with LA and one with GA). There were no other exclusion criteria in this study. Thus, 90 patients diagnosed as incarcerated inguinal hernia and underwent emergency inguinal hernia repair were included in the final analysis. There were 32 patients (20 men and 12 women) in LA group and 58 patients (45 men and 13 women) in GA group. All patients received regularly follow-up (7 and 30 days after surgery) and 87 patients received the regularly follow-up of 90 days after the surgery (including 26 patients followed by telephone).

### General condition

General condition of all patients was shown in Table 1. The mean age of patients in GA group was 77.4 ± 12.7 years (range from 30 to 94), and 79.3 ± 17.9 years (range from 30 to 99) in LA group (*P* = 0.554). 15.6 % of all patients had the duration of symptoms shorter than 6 h; 23.3 % between 6 to 12 h; 17.8 % between 12 to 24 h; 43.3 % longer than 1 day. The duration of symptoms in LA group was longer than that in GA group (*P* = 0.013). LA group had one case of recurrent hernia and nine cases were in group GA. LA group had a higher ASA Grade than that of GA group (*P* = 0.007).

**Table 1:** Characteristics of patients

	LA (*n* = 32)	GA (*n* = 58)	*P*
Mean age (SD) in years	79.31(17.94)	77.38(12.71)	0.554
Sex			0.126
Male	20(62.5 %)	45(77.6 %)	
Female	12(37.5 %)	13(22.4 %)	
BMI			0.223
Mean (SD)	23.10(3.45)	24.29(3.20)	
Site of hernia			0.142
Right	15(46.9 %)	31(53.4 %)	
Left	17(53.1 %)	27(46.6 %)	
Type of hernia			0.002*
Primary	31(96.9 %)	49(84.5 %)	
Recurrent	1(3.1 %)	9(15.5 %)	
ASA Grade			0.014*
II	17(53.1 %)	42(72.4 %)	
III	11(34.3 %)	16(27.6 %)	
IV	4(12.6 %)	0(0 %)	
Duration			0.013*
<6 h	2(6.25 %)	12(20.7 %)	
6-12 h	8(25 %)	13(22.4 %)	
12-24 h	2(6.25 %)	14(24.1 %)	
>24 h	20(62.5 %)	19(32.8 %)	

* *P* < 0.05

### Surgical procedure

One case with bowel resection was performed under LA. In GA group, seven cases (included one converted from LA) had bowel resection. In LA group, 31 patients underwent mesh repairs, except the cases with bowel resection. In GA group, 50 patients received mesh repairs while eight received tissue repairs (included all seven cases with bowel resection). In two cases of LA group and two cases of GA group, the incarcerated hernia sacs returned spontaneously during the operation.

### Postoperative recovery

Patients in LA group had a shorter hospital stay than those in GA group (4.31 ± 1.58 vs 5.88 ± 2.82 days, *P* = 0.001). LA group also had shorter ICU stay, time to eat, time to ambulation and less total costs. All the differences were statistical significant and presented in Table 2.

**Table 2:** Complications of GA and LA group

	LA (*n* = 32)	GA (*n* = 58)	*P*
Time in hospital	4.31 ± 1.575	5.88 ± 2.816	0.001*
Time in ICU	0.03 ± 0.177	0.34 ± 1.085	0.035*
Time to eat	1.28 ± 0.581	2.28 ± 1.121	0.000*
Time to ambulation	2.03 ± 1.031	3.26 ± 1.528	0.000*
Total cost	8836.59 ± 2436.673	13236.43 ± 5461.154	0.000*
Wound infection	4 (12.5 %)	9 (15.5 %)	0.765
Urinary retention	1 (3.1 %)	6 (10.3 %)	0.414
Postoperative hydrocoele	2 (6.3 %)	6 (10.3 %)	1.000
Respiratory complications	2 (6.3 %)	14 (24.1 %)	0.044*
Cardiovascular complications	1 (3.1 %)	12 (20.7 %)	0.027*
MODS	0 (0 %)	1 (1.7 %)	1.000
Death in 30 days	0 (0 %)	1 (1.7 %)	1.000

*MODS* multiple organ disfunction syndrome

* *P* < 0.05

### Complications

A summary of complications of two groups was presented in Table 2. LA group had fewer respiratory and cardiovascular complications than the GA group. 87 patients received a 3-month regular follow-up after discharge. No recurrence was observed.

## Discussion

GA is widely used in hernia repair. It can provide the surgeon with optimal operating condition in terms of patient immobility and a satisfactory muscular relaxation [10]. GA has been proved to be a good choice in hernia repair surgery, both in elective and emergency operations. In elective operations, LA has showed a lower risk compared to GA [11, 12]. But the role of LA in emergency inguinal hernia repair is still controversial [8, 9, 13, 14].

Intraoperative pain seems to be the most common reason for dissatisfaction with LA. One report demonstrated that infiltration was painful and 8.5 % of patients experienced pain intraoperatively [15]. Incarcerated hernia always has a complicated anatomy, pain and tension may result in an unsatisfactory muscular relaxation. In such condition, many surgeons worried that emergency operations might overdose on local anesthetics. With improvement in surgical technique, surgeons became knowledgeable regarding the anatomy of the inguinal region as well as the application of LA. The indication of LA application was widened step by step. In this study, we tried to apply LA in emergency incarcerated hernia repair and evaluate its feasibility.

LA of inguinal hernia repair reported in the literature include “one step procedure” LA [16], ultrasound-guided transversus abdominis plane block and unilateral paravertebral block [17]. The argument about the “best” type and ratio of the anesthetics has never stopped [18–21]. In this study, we followed a “step by step technique”, which was first reported by Amid in 1994 [22]. In two cases, the surgeon even finished the contralateral inguinal hernia repair with the given mixture. Our study performed 32 cases in LA successfully (33 in total). There was only one case converted to GA, in which bowel resection was performed. So LA is feasible for emergency incarcerated hernia if the possibility of bowel resection is limited. In other two cases, we observed that the contents of hernia sacs returned spontaneously, which showed that LA might also have the effect of tissue relaxation.

In our study, the patient characteristics between two groups were different. Patients in LA group had a longer duration of symptoms and a higher ASA grade. This is because the group was nonrandomized. Surgeons and anesthetists tended to group the patients with serious comorbid illness in LA to avoid risk of complications of GA. Meanwhile patients who suffered longer incarcerated time and likely got bowel resection might receive GA according to medical staff. Even in this situation, our study still revealed significant advantages for LA in postoperative cardiopulmonary complications. LA can allow patients to start eating earlier and have early ambulation, therefore reducing the time of nutritional support and avoiding postoperative cardiopulmonary infection and finally reducing hospital costs and hospital stays. Our study found that patients in LA group had advantages compared to those in the GA group on ICU residence time, total hospital stay and total hospital cost. It was demonstrated that inguinal hernia repair in incarcerated hernia patients is feasible under LA.

LA also has its disadvantages. First, intraoperative pain is one of the most common reasons for dissatisfaction with LA. In our study, LA was performed not only by the surgeon but also monitored by an anesthetist, who played an important part in keeping safety of the operation. In some difficult cases, extra analgesic or sedative drugs given intravenously by the anesthetist can improve the success rate of LA. Second, whether meshes can be applied in the emergency repair surgery under LA is still inconclusive [23, 24]. Our previous report showed that prosthetic mesh could be used if no bowel resection performed, duration of symptoms less than 24 h and fluid hernia sac was clear [25]. In this study, tension-free repair with meshes was applied in 81 cases based on the above principles. Third, H. Kehlet reported that local anesthesia was one of the risk factors for recurrence after groin hernia repair [26]. Although no recurrence case was observed in our study with a 3-month follow-up, a longer time follow-up period is necessary.

Our study has its limitations. For this is a retrospective study, the lack of randomization is a major problem. We hope to have more conclusive results in further studies, which can be well-designed randomized controlled trials with larger sample size.

## Conclusion

LA could provide effective anesthesia and patient safety in emergency inguinal hernia repair, especially when the possibility of bowel resection is limited. Further prospective studies are needed to confirm this finding.

## Consent

Written informed consent was obtained from the patient for the publication of this report and any accompanying images.

## References

[1] Lomanto D, Cheah WK, Faylona JM (2015). Inguinal hernia repair: toward Asian guidelines. Asian J Endosc Surg.

[2] Kulah B, Duzgun AP, Moran M (2001). Emergency hernia repairs in elderly patients. Am J Surg.

[3] Kjaergaard J, Bay-Nielsen M, Kehlet H (2010). Mortality following emergency groin hernia surgery in Denmark. Hernia.

[4] Rosenberg J, Bisgaard T, Kehlet H (2011). Danish Hernia Database recommendations for the management of inguinal and femoral hernia in adults. Dan Med Bull.

[5] Nordin P, Zetterstrom H, Carlsson P (2007). Cost–effectiveness analysis of local, regional and general anaesthesia for inguinal hernia repair using data from a randomized clinical trial. Br J Surg.

[6] O’Dwyer PJ, Serpell MG, Millar K (2003). Local or general anesthesia for open hernia repair: a randomized trial. Ann Surg.

[7] Reece-Smith AM, Maggio AQ, Tang TY (2009). Local anaesthetic vs. general anaesthetic for inguinal hernia repair: systematic review and meta-analysis. Int J Clin Pract.

[8] David S, Martin K, et al. Groin hernia guidelines, Association of Surgeons of Great Britain and Ireland, Available at: http://www.asgbi.org.uk/download.cfm?docid=21BC920F-290E-4CDE-B415CA77DFF1E0A0

[9] Simons MP, Aufenacker T, Bay-Nielsen M (2009). European Hernia Society guidelines on the treatment of inguinal hernia in adult patients. Hernia.

[10] Sanjay P, Woodward A (2007). Inguinal hernia repair: local or general anaesthesia?. Ann R Coll Surg Engl.

[11] Amato B, Compagna R, Della Corte GA (2012). Feasibility of inguinal hernioplasty under local anaesthesia in elderly patients. BMC Surg.

[12] Nordin P, Zetterstrom H, Gunnarsson U, Nilsson E (2003). Local, regional, or general anaesthesia in groin hernia repair: multicentre randomised trial. Lancet.

[13] Nordin P, Hernell H, Unosson M (2004). Type of anaesthesia and patient acceptance in groin hernia repair: a multicentre randomised trial. Hernia.

[14] Teasdale C, McCrum AM, Williams NB (1982). A randomised controlled trial to compare local with general anaesthesia for short-stay inguinal hernia repair. Ann R Coll Surg Engl.

[15] Kurmann A, Fischer H, Dell-Kuster S (2015). Effect of intraoperative infiltration with local anesthesia on the development of chronic pain after inguinal hernia repair: a randomized, triple-blinded, placebo-controlled trial. Surgery.

[16] Dabić D, Perunicić V, Marić B (2012). “One step procedure” local anaesthesia for inguinal hernia repair in ambulatory surgery conditions--district general hospital experience. Acta Chir Iugosl.

[17] Bhattacharya P, Mandal MC, Mukhopadhyay S (2010). Unilateral paravertebral block: an alternative to conventional spinal anaesthesia for inguinal hernia repair. Acta Anaesthesiol Scand.

[18] Compagna R, Vigliotti G, Coretti G (2012). Comparative study between Levobupivacaine and Bupivacaine for hernia surgery in the elderly. BMC Surg.

[19] Milone M, Di Minno MN, Musella M (2013). Outpatient inguinal hernia repair under local anaesthesia: feasibility and efficacy of ultrasound-guided transversus abdominis plane block. Hernia.

[20] Kulacoglu H, Ergul Z, Esmer AF (2011). Percutaneous ilioinguinal-iliohypogastric nerve block or step-by-step local infiltration anesthesia foringuinal hernia repair: what cadaveric dissection says?. J Korean Surg Soc.

[21] De Sá Ribeiro FA, Padron F, Castro TD (2010). Inguinal hernia repair with local anesthesia in the outpatient. Rev Col Bras Cir.

[22] Amid PK, Shulman AG, Lichtenstein IL (1996). Open “tension-free” repair of inguinal hernias: the Lichtenstein technique. Eur J Surg.

[23] Paajanen H, Varjo R (2010). Ten-year audit of Lichtenstein hernioplasty under local anaesthesia performed by surgical residents. BMC Surg.

[24] De Martino A, Testi W, Cirianni D (2007). Incarcerated inguinal hernia in elderly: personal tension-free hernioplastic technique. Ann Ital Chir.

[25] Yang L, Wang H, Liang X (2015). Bacteria in hernia sac: an important risk fact for surgical site infection after incarcerated hernia repair. Hernia.

[26] Kehlet H, Bay-Nielsen M (2008). Local anaesthesia as a risk factor for recurrence after groin hernia repair. Hernia.

